# Letter in response to Laing et al., “An evaluation of radiation therapy patient body mass index trends and potential impact on departmental resource planning”

**DOI:** 10.1002/jmrs.684

**Published:** 2023-05-14

**Authors:** Amanda Bolderston, Megan Brydon

**Affiliations:** ^1^ University of Alberta Edmonton Alberta Canada; ^2^ IWK Health Centre Halifax Nova Scotia Canada

## Abstract

“An evaluation of radiation therapy patient body mass index trends and potential impact on departmental resource planning” by Laing et al. The authors’ comment that research into the experiences of larger bodied patients should focus on compassionately improving care for this patient population rather than framing large‐bodied patients as a burden or problem, and should include commentary on the effects of weight‐bias in the healthcare system.

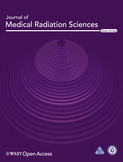

We read with interest the recent publication by Laing et al., “An evaluation of radiation therapy patient body mass index trends and potential impact on departmental resource planning”.^1^

We are currently conducting a scoping review of medical radiation sciences research associated with large‐bodied patients. This review revealed many papers, such as this one, written with a deficit based lens. Deficit based research in healthcare focuses on the “burden” or “challenges” of a particular population. Language characterising patients as “challenging”, “difficult”, “inconvenient” or “embarrassing” may be used.^2^ Repeatedly framing health issues as a problem or inconvenience in the context of a specific population (in this case, large patients) can contribute to stigmatisation and practitioner bias. This has far‐reaching negative consequences for the affected communities.

Weight bias is endemic in healthcare and has deleterious effects on patients' ability to access and navigate the healthcare system.^3^ Weight bias situates large bodies as a health crisis and personal failing. Patients may delay or avoid care because of previous negative experiences, embarrassment or mistrust. Healthcare providers often exhibit weight‐bias and may discriminate against large‐bodied patients.^4^

We would like to respectfully discuss four specific examples from this paper of deficit‐based framing and weight‐bias:

1. The authors refer to some well‐known practicalities in treating large‐bodied patients, such as physical equipment limits. They discuss the safe working loads of various immobilisation equipment. The authors conclude that “excessive load” related to large patients may increase the possibility of equipment malfunction and that equipment should be checked regularly for wear and tear. We suggest that this is speculative at best, and that regular equipment checks, and maintenance are important regardless of patient size. We feel the authors overlooked the fact that radiation therapy departments should advocate for manufacturers to improve their equipment to accommodate patients, rather than viewing large patients as “excessive load”.
2. The paper references two studies^5^, ^6^ where the treatment of several patients was compromised due to a lack of suitable equipment (e.g. couch weight limitations). The authors concluded that “these papers hint that departments are experiencing equipment limitations when it comes to bariatric patients, despite it not being widely reported” (n.p) and patients may need to be referred to bariatric‐capable departments. These examples further emphasise that the lack of suitable equipment, techniques, and equitable access are the problems, not the patients themselves.
3. The authors reference two older papers (from 2009 and 2012) to indicate that large patients are harder to set up using external landmarks, are more prone to set up errors, and may need more image verification.^7^, ^8^ The authors could have included current literature on adaptive treatment strategies such as daily image verification. This is now common practice for many patient populations and can mitigate localisation uncertainties.^9^, ^10^
4. The authors cite a recent qualitative publication by Winters and Poole where radiation therapists were asked what “challenges” they associated with large‐bodied patients. The authors reported that, “obese patients took longer to mount and dismount the bed…(which)…further increased time delays on the treatment unit” (Ref. 11, p. 161). Regardless of the participants' perceptions, blaming patients for workload and treatment delays is problematic. This stance again centres the patient as the problem, rather than the lack of time allocated to adequately care for patients with various needs. Many patients take longer to care for, not just those with larger bodies.

We are sure the authors had the best intentions. However, future research should focus on compassionately improving care for this patient population rather than framing large‐bodied patients as a burden or problem.
